# A strategy to identify a ketoreductase that preferentially synthesizes pharmaceutically relevant (*S*)-alcohols using whole-cell biotransformation

**DOI:** 10.1186/s12934-018-1036-2

**Published:** 2018-12-03

**Authors:** Saiful F. Haq, Anirudh P. Shanbhag, Subbulakshmi Karthikeyan, Imran Hassan, Kannan Thanukrishnan, Abhishek Ashok, Sunilkumar Sukumaran, S. Ramaswamy, Nagakumar Bharatham, Santanu Datta, Shalaka Samant, Nainesh Katagihallimath

**Affiliations:** 1 Anthem Biosciences Pvt. Ltd, Bengaluru, India; 2 Bugworks Research India, Pvt. Ltd, Bengaluru, India; 3 Centre for Pharmaceutical Biotechnology, University of Illinois Chicago, Chicago, USA; 4 Shasun Research Center, Chennai, India; 5 Institute for Stem Cell Biology and Regenerative Medicine, Bengaluru, India; 6 Centre for Cellular and Molecular Platforms, Bengaluru, India; 7 Department of Biophysics, Molecular Biology and Bioinformatics, University of Calcutta, Kolkata, India; 8 PerkinElmer, Bengaluru, India

**Keywords:** Biotransformation, Chiral alcohol synthesis, Short chain dehydrogenase, Medium chain dehydrogenase, Ketoreductase

## Abstract

**Introduction:**

Chemical industries are constantly in search of an expeditious and environmentally benign method for producing chiral synthons. Ketoreductases have been used as catalysts for enantioselective conversion of desired prochiral ketones to their corresponding alcohol. We chose reported promiscuous ketoreductases belonging to different protein families and expressed them in *E.* *coli* to evaluate their ability as whole-cell catalysts for obtaining chiral alcohol intermediates of pharmaceutical importance. Apart from establishing a method to produce high value (*S*)-specific alcohols that have not been evaluated before, we propose an in silico analysis procedure to predict product chirality.

**Results:**

Six enzymes originating from *Sulfolobus* *sulfotaricus, Zygosaccharomyces* *rouxii*, *Hansenula* *polymorpha*, *Corynebacterium *sp. ST-10, *Synechococcus* sp. PCC 7942 and *Bacillus *sp. ECU0013 with reported efficient activity for dissimilar substrates are compared here to arrive at an optimal enzyme for the method. Whole–cell catalysis of ketone intermediates for drugs like Aprepitant, Sitagliptin and Dolastatin using *E.* *coli* over-expressing these enzymes yielded (*S*)-specific chiral alcohols. We explain this chiral specificity for the best-performing enzyme, i.e., *Z.* *rouxii* ketoreductase using in silico modelling and MD simulations. This rationale was applied to five additional ketones that are used in the synthesis of Crizotinib, MA-20565 (an antifungal agent), Sulopenem, Rivastigmine, Talampanel and Barnidipine and predicted the yield of (*S*) enantiomers. Experimental evaluation matched the in silico analysis wherein ~ 95% (*S*)-specific alcohol with a chemical yield of 23–79% was obtained through biotransformation. Further, the cofactor re-cycling was optimized by switching the carbon source from glucose to sorbitol that improved the chemical yield to 85–99%.

**Conclusions:**

Here, we present a strategy to synthesize pharmaceutically relevant chiral alcohols by ketoreductases using a cofactor balanced whole-cell catalysis scheme that is useful for the industry. Based on the results obtained in these trials, *Zygosaccharomyces* *rouxii* ketoreductase was identified as a proficient enzyme to obtain (*S*)-specific alcohols from their respective ketones. The whole–cell catalyst when combined with nutrient modulation of using sorbitol as a carbon source helped obtain high enantiomeric and chemical yield.

**Electronic supplementary material:**

The online version of this article (10.1186/s12934-018-1036-2) contains supplementary material, which is available to authorized users.

## Background

Chiral alcohols are essential building blocks in the synthesis of pharmaceutical molecules and fine chemicals [1–3]. Asymmetric reduction of prochiral ketones using enzymatic biotransformation is useful for synthesizing products of enantiomeric purity. In contrast to chemical methods that often do not comply with the principles of green chemistry, biocatalysis offers numerous advantages, such as mild and environmentally benign conditions and remarkable chemo-, regio- and stereoselectivity [4, 5]. It often facilitates the circumvention of arduous syntheses routes that require multiple protection and deprotection steps [6, 7]. Besides, they can provide an advantage of obtaining up to 100% theoretical yield with over 99% enantioselectivity [8]. Biocatalysis for prochiral ketone reduction could either employ a whole-cell system or a purified enzyme preparation [4]. The use of whole-cells offers the advantage of being a simple and low-cost catalyst preparation compared to purified enzymes that are generally expensive due to the need of protein purification, their diminished activity under process conditions, insufficient stability and vulnerability to the substrate and product inhibitions [9]. Enzymes are often more stable within the cellular milieu, as microorganisms can insulate them from harsher environment [10]. Enzymatic reduction of ketones is performed with a stoichiometrically higher proportion of cofactors (NADH or NADPH) as that of the substrate which adds to the cost of synthesis. Cells provide a basal capacity for cofactor regeneration through the reduction of NAD+ and NADP+ in central metabolic pathways [11]. The use of whole-cell ketoreductase system also precludes the costly and tedious process of separating the enzymes from the reaction mixture as the products are harvested from media. To identify an efficient enzyme for the process of ketone reduction, we considered two super families of ketoreductases that can convert a broad range of prochiral ketones namely, the medium-chain (MDR) and short-chain (SDR) dehydrogenases/reductases (nineteen enzymes as listed in Table 1 and references therein [12–28].

**Table 1:** Prospecting ketoreductases from published literature

Protein name*	Protein superfamily and family	Amino acids	Organism
***FabG***	**NADB Rossmann BKR like**	**249**	***S. elongatus*** [12]
*Scored*	NADB Rossmann SDRc	263	*S. coelicolor* [13]
*Cmafph*	NADB Rossmann MDH Like	254	*C. maris* [14]
*LbRADH*	NADB Rossmann 3β-17β HSD Like	257	*L. brevis* [15]
*CpScrII*	NADB Rossmann MDH Like	279	*P. stipites* [16]
*Pscr*	NADB Rossmann MDH Like	282	*P. stipites* [17]
***Hketo***	**NADB Rossmann MDH Like**	**273**	***H. polymorpha*** [18]
*CmagS1*	NADB Rossmann MDH Like	283	*C. magnoliae* [19]
*Kacr1*	NADB Rossmann MDH Like	292	*K. lactis* [20]
***BYueD***	**NADB Rossmann SPR like SDR**	**243**	***B.*** **subtilis** [21]
*SSAkr2*	NADB Rossmann AR SDR Like	323	*S. salmonicolor* [22]
***ZRK***	**NADB Rossmann AR SDR Like**	**338**	***Z. rouxii*** [23]
*LKadh*	MDR FDH Like ADH3	347	*L. kefir* [24]
*HVadh2*	MDR FDH Like ADH3	349	*H. volacanii* [25]
*HVadh1*	MDR ADH6	353	*H. volacanii* [25]
***SsADH***	**MDR Arabinose DH like**	**347**	***S.*** **solfataricus** [26]
*SaADHRC3*	MDR Arabinose DH like	347	*S. solfataricus* [27]
***PAR***	**MDR Arabinose DH like**	**385**	***Corynebacteirum*** **sp.** [28]
*SSAkr1*	NADB Rossmann AR SDR Like	343	*S. salmonicolor* [22]

Six ketoreductases highlighted in bold were chosen from a set of 19 enzymes based on their presence in different genera and molecular weight, showing varying optomer specific catalysis based on literature

* The nomenclature used for the ketoreductases are from the references denoted in parenthesis

An often-used technique for engineering enzymes to obtain desired properties of enzyme promiscuity or substrate specificity is DNA shuffling or random mutagenesis [29]. Else, numerous alcohol dehydrogenases [30] or SDRs-Carbonyl reductases [31] are screened to obtain the best enzyme that is useful for a specific substrate. Although these methods have been successfully used they are essentially a ‘black-box’ approach to obtain an optimal solution. Even minor changes in substrate structure or alignment of amino-acids in the active site drastically alters the specific activity of the enzyme [32]. We demonstrate a rational approach for choosing dehydrogenases that not only circumvents the arduous process of high-throughput screening of shuffled enzymes but aids in making a rational choice of enzymes from different families [33]. To determine the ability of enantioselective conversion, we tested six ketoreductases (highlighted in bold in Table 1) against three high value prochiral pharmaceutical intermediates. We observed that only the ketoreductase *Zygosaccharomyces* *rouxii* short chain dehydrogenase/reductase (ZRK) had the required catalytic characteristics that produced the near total (*S*)-enantioselectivity with a robust chemical yield under whole cell bio-transformation. We further investigated the probable reasons for the aforementioned stereo-selective product formation and identified 5 other ketones of pharmaceutical importance that could be catalyzed to produce high value (*S*)-enantiomers. Binding mode of the ketones included in the current study were analyzed by molecular docking and the predictions were further validated by molecular dynamics simulations of ZRK: substrate complex. The in silico predictions have been validated by experimentation.

## Results and discussion

### Ketoreductase selection, their over-expression and solubility in *E. coli* BL21(DE3)

**Fig. 1:**
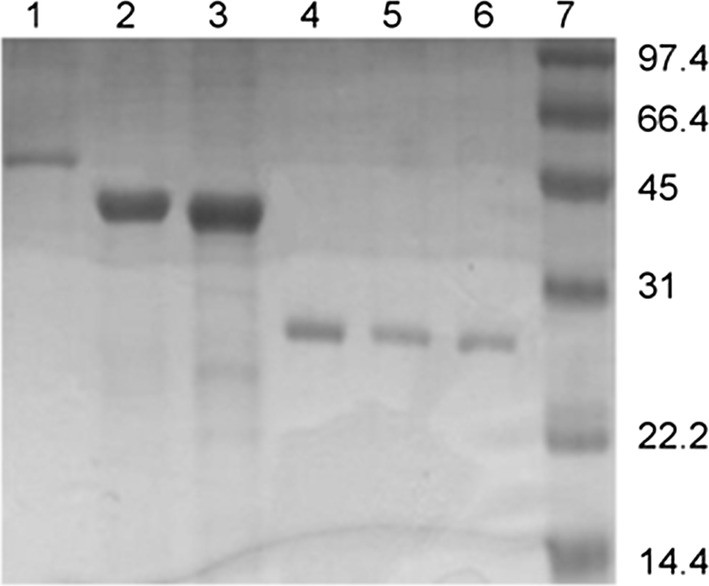
SDS PAGE of all the isolated His-tagged ketoreductases. Lanes 1. *Corynebacterium* strain ST-10 phenylacetaldehyde reductase (PAR), 2. *Sulfolobus sulfotaricus* alcohol dehydrogenase (SsADH), 3. *Zygosaccharomyces rouxii* SDR (ZRK), 4. *Hansenula polymorpha* ketoreductase (Hketo), 5. *Bacillus subtilis* yueD (BYueD), 6. 3-oxoacyl-[acyl-carrier-protein] reductase (FabG) and 7. low range molecular weight marker

It is a challenge to design an ideal ketoreductase for maximum chemical yields and enantioselectivity based on the enzyme/substrate structure or sequence alone [33]. Literature studies indicate that short-chain and medium-chain dehydrogenases/reductases (SDRs and MDRs) have high structural similarity despite dissimilar protein sequences. MDRs are 300–400 amino acid long, zinc containing dimeric enzymes. They are well-known reducing agents for non-polar metabolites and act as phase-I detoxification agents [34, 35] indicating a high substrate promiscuity, and this is a feature that is useful to convert multiple substrates. SDRs are also dehydrogenases that are 200–300 amino acids long and exist as monomers and have a different mechanism of catalysis and active site [35]. Therefore, the selection of enzymes was based on taxonomic divergence, molecular weight and their reported enantioselectivity of common ketones in the literature. Six ketoreductases belonging to Archaea/Bacteria/Eukarya namely, *Sulfolobus* *sulfotaricus* alcohol dehydrogenase (ADH) (SsADH), SDRs *Zygosaccharomyces rouxii* SDR (ZRK) and *Hansenula* *polymorpha* DL-1 peroxisomal 2,4-dienoyl-CoA reductase (Hketo) and lastly, *Corynebacterium* strain ST-10 phenylacetaldehyde reductase (PAR), *Synechococcus *sp. PCC 7942 3-ketoacyl-[acyl-carrier-protein] reductase (FabG) and *Bacillus *sp. ECU0013 ADH (ByeuD) were chosen, (highlighted in bold in Table 1). *E. coli* was selected as the organism to express the recombinant ketoreductases given its ease of growth to obtain large biomass and well-understood physiology. The enzymes were over-expressed using the T7 expression system consisting of the respective ketoreductase–pET28a plasmids and the *E. coli* BL21(DE3) strain. It is essential for the proteins to be soluble in vivo for efficient whole-cell catalysis. Therefore, the expression of the enzyme was verified by isolating the His-tagged proteins from cytosolic fractions and visualizing on a Coomassie stained SDS–PAGE (Fig. 1). The over-expression of the desired ketoreductase also helps reduce side reactions from other competing enzymes in the bacterial cell.

### ZRK shows the best activity towards a variety of ketones amongst the compared ketoreductases

Six ketoreductases are compared for catalyzing three pro-pharmaceutical chiral compounds 3, 6 and 8 as substrates in Table 2. SsADH, PAR and ByeuD that converted below 30% for all substrates were deemed as low yielding reactions. FabG and Hketo showed moderate conversions, 30–60% of Sitagliptin intermediate (3-oxo-4-(2,4,5-trifluoro-phenyl) butyric acid methyl ester) and Dolastatin intermediate (2-phenyl-1-thiazol-2-yl-ethanone). However, its yield was less than 2% in the case of Aprepitant intermediate [1- (3,5-Bis-trifluoromethyl-phenyl)-ethanone]. ZRK stands out with > 98% yield for Sitagliptin and Dolastatin intermediates and 22% for the Aprepitant intermediate. HPLC analysis revealed an excess of (*S*)-specific alcohol for the three ketones, data for ZRK shown in Table 3. It should be noted that the Aprepitant intermediary of interest is an (*R*)-enantiomer but the enzyme produces an (*S*)-isomer and hence has been included in the list of substrates. We hypothesised that there underlies a specific mode of substrate binding that results in (S)-specific conversion. Elucidating the mode of substrate binding could help exploit the ketoreductases for obtaining specific chiral synthons. The most efficient enzyme in the current study i.e., ZRK, was selected for evaluation by in silico modelling and docking experiments.

**Table 2:** Comparison of alcohol yields for commercially relevant ketones using six ketoreductases

Prochiral ketone	Alcohol yield (%)					
	SsADH	ByeuD	FabG	Hketo	PAR	ZRK
Aprepitant ketone intermediate (compound 3)	0.8	7	0	1.32	0	22.6
Sitagliptin ketone intermediate (compound 8)	9.6	29	38	87	18	98
Dolastatin ketone intermediate (compound 6)	2.98	26	31.8	41	2	>99

ZRK was found to be the best catalyst among the chosen enzymes. The substrates used are as follows, aprepitant ketone intermediate: 1-(3,5-bis-trifluoromethyl-phenyl)-ethanone, Sitagliptin ketone intermediate: 3-oxo-4-(2,4,5-trifluoro-phenyl) butyric acid methyl ester and dolastatin ketone intermediate: 2-phenyl-1-thiazol-2-yl-ethanone

**Table 3:** Sorbitol is better than glucose as carbon source

Ketone intermediate	Alcohol yield *%*			
	PBS	PBS with glucose	PBS with sorbitol	(*S*)-enantiomer
Talampanel ketone intermediate (compound 1)	1.25	79	87	90
MA-20565 ketone intermediate (compound 5)	2.75	37	91	100
Dolastatin ketone intermediate (compound 6)	5	23	100	96

Whole-cell *E.* *coli* expressing ZRK in sorbitol containing media gives higher alcohol yields than in glucose (average yields from three experiments reported). The substrates used are as follows, talampanel ketone intermediate: 3,4-methylenedioxyphenyl acetone, MA-20565 ketone intermediate: 3–trifluoromethyl acetophenone and dolastatin ketone intermediate: 2-Phenyl-1-thiazol-2-yl-ethanone

### Sorbitol is a preferred carbon source compared to glucose for cofactor enrichment

During biotransformation, *E. coli* has a finite concentration of NAD(P)H, which would restrict the conversion of the substrate ketone. Regeneration of cofactors is essential for high catalytic potential [36]. In-vitro enzyme mediated cofactor regeneration methods employ either an enzyme or substrate coupled approach. These strategies typically use glucose/formate dehydrogenase to catalyze the reduction of NAD(P) [37]. A non-genetic means of enhancing cofactors is by modifying the external environment by using carbon sources with a lower oxidation state [38]. Glucose, the commonly used carbon source for fermentation has an oxidation state of 1. Other carbon sources like xylose, fructose and sorbitol have an oxidation state of 0, + 1 and − 1 respectively [39]. Sorbitol with the lowest oxidation state would be the preferred carbon source to be evaluated.

**Fig. 2:**
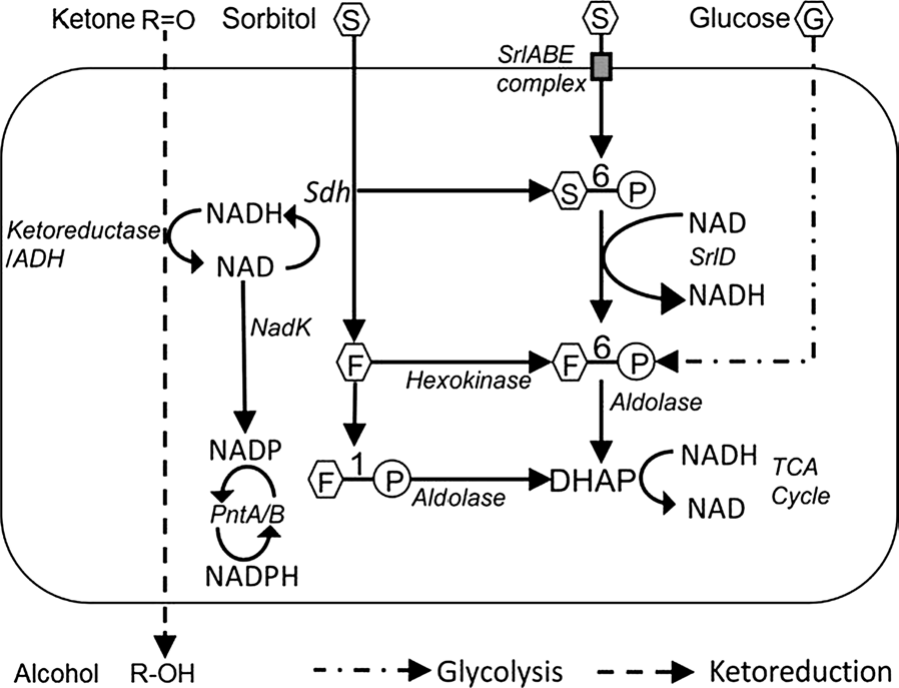
Sorbitol metabolism in *E. coli.* A model illustrating the biochemical pathways involved in the production of additional reduced cofactors NAD(P)H by introduction of sorbitol as the carbon source instead of glucose in whole-cell biotransformation

In bacteria, sorbitol metabolism produces NADH using two separate pathways. It is catalyzed by sorbitol dehydrogenase (*Sdh*) to form fructose or as in *E. coli*, the sorbitol permease complex (*SrlA/B/E*) helps in the uptake and phosphorylation of sorbitol into sorbitol-6-phosphate. Subsequently, it is oxidized by sorbitol-6-phosphate dehydrogenase (*SrlD*) into fructose-6-phosphate [40, 41] with a concomitant release of NADH. This fructose/fructose–6–phosphate proceeds towards the glycolytic pathway (Fig. 2). We hypothesize that this additional NADH enhances the pool of cofactors available for the ketoreductases to convert the ketone to their respective alcohol. NADH and NADPH are inter-converted during cellular energy deficit by nadK (NAD Kinase) which converts NAD^+^ into NADP^+^. It is subsequently reduced to NADPH by the membrane bound or soluble pyridine nucleotide transhydrogenase (*PntA/B*) [42] as illustrated in Fig. 2. Enhanced alcohol production was observed in cells utilizing sorbitol instead of glucose as the carbon source during biotransformation (Table 3).

### In silico ZRK model with other relevant ketones showed a potential (*S*)-enantiomer producing enzyme

Homology modelling and molecular docking provide reasonable information about substrate interaction with the catalytic site and also help deduce reasons for stereo-selectivity [43–45]. Some of these studies incorporate stereochemistry rules such as Prelog and anti-Prelog [44] and state that combining these rules with binding orientations provide high success in predicting the product enantiomer [44]. Understanding the mode of substrate binding could help us exploit these enzymes for obtaining specific product enantiomers. To discern the high (*S*)-enantiomeric excess (%EE), we considered using similar methods in probing the substrate binding mode and mechanism of ZRK for further application to other ketones.

#### Homology modelling of ZRK

**Fig. 3:**
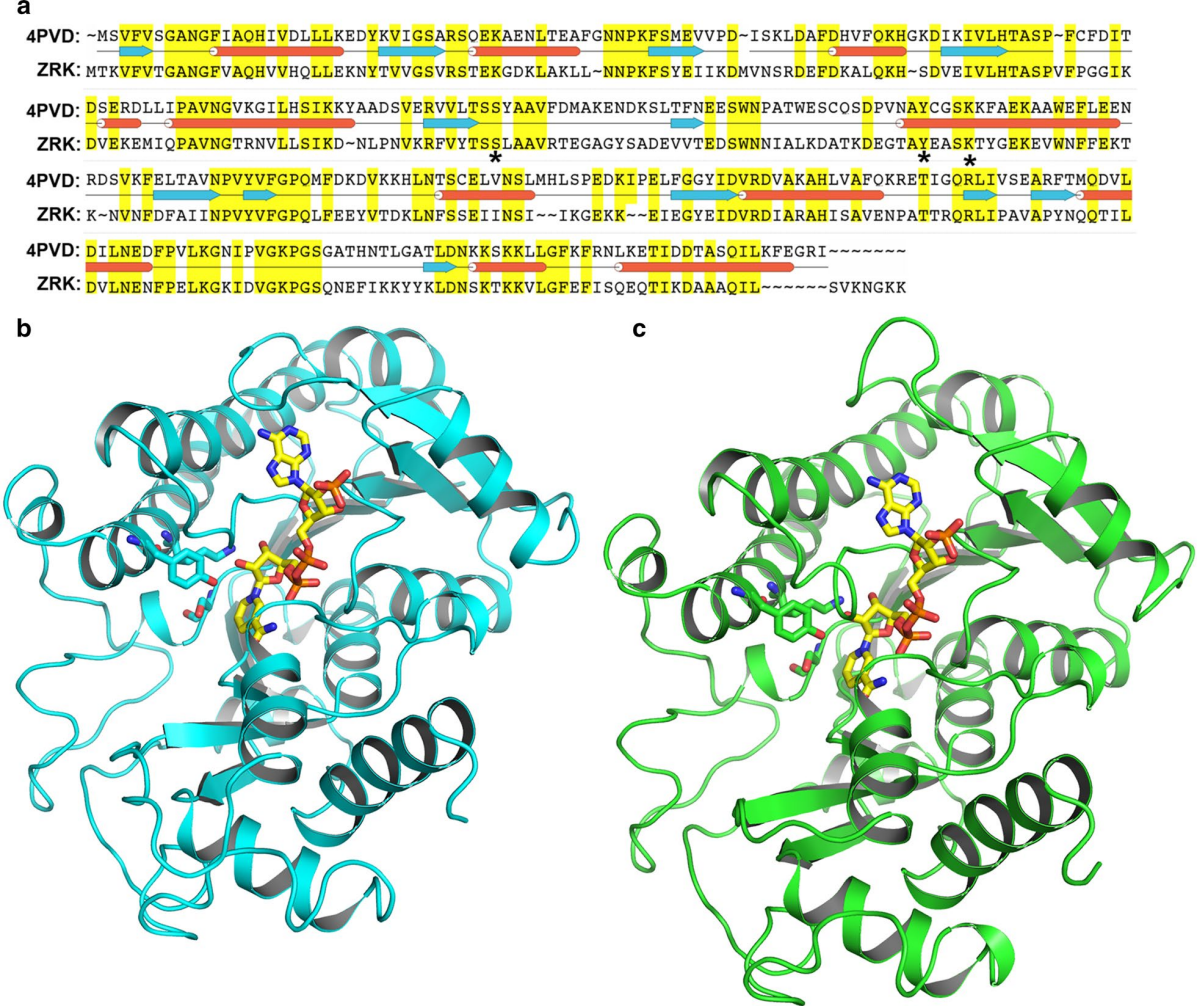
Modelling of ZRK for MD simulations. The sequence alignment between *Zygosaccharomyces rouxii* ketoreductase (ZRK) and yeast methylglyoxal/isovaleraldehyde reductase Gre2 (4PVD) sequences which is utilized to build homology model of ZRK. **a** The catalytic triad is highlighted with an asterisk in the sequence alignment panel. **b** Cartoon representation of the template structure. **c** Cartoon representation of ZRK. NADPH is represented as yellow sticks and catalytic triad highlighted with sticks in both structures

ZRK was modelled using yeast methyl glyoxal/isovaleraldehyde reductase Gre2 [PDB ID: 4PVD] [45] as a template that has 44% homology (Fig. 3a) using SWISS-MODEL. PROCHECK was used to validate the 3D structure; it showed ~ 88% of the residues lie in the most favored region (Fig. 3b) compared to ~ 91% residues in the template (Fig. 3c). The Root Mean Square Deviation (RMSD) between Cα atoms of the template and the target structures was 0.7 Å. Structural and sequential information, by comparison, showed the catalytic triad serine, tyrosine and lysine (important for ketone to alcohol reduction) [46] was conserved in the ZRK model.

#### Substrate binding mode evaluation shows (*S*)-enantiomer oriented reduction

**Fig. 4:**
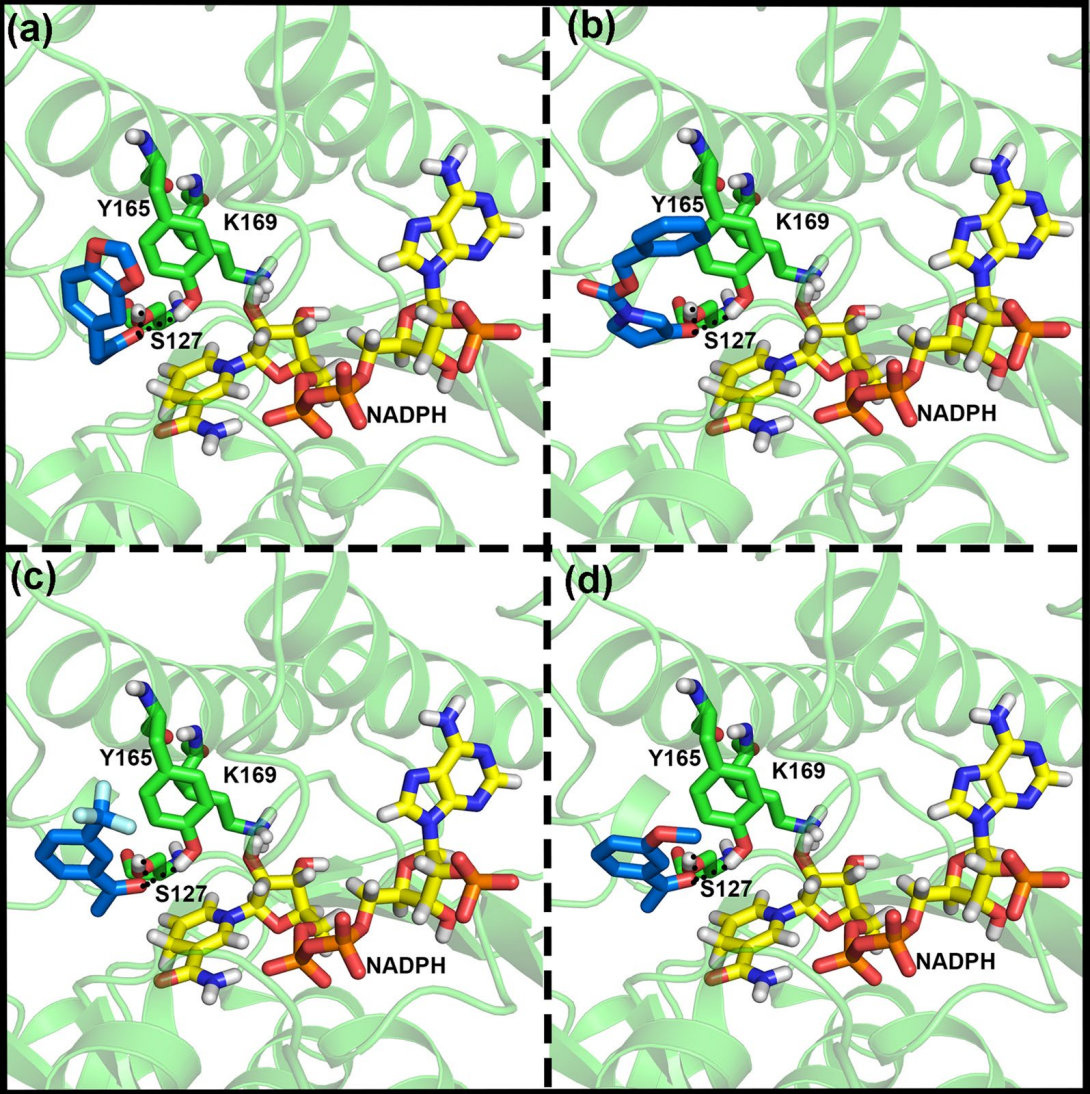
Binding mode of substrate moleucles. Molecular docking predicted binding interaction (depicted in dashed black lines) modes for compound 1**a**, compound 2**b**, compound 3**c**, and compound 7**d**. Substrates are represented with blue sticks, catalytic residues and NADPH are shown as green and yellow sticks respectively. Hydrogen bonds between substrate and triad residues are depicted with broken lines

Molecular docking was used to predict substrate binding to the ZRK model  [47]. Additional pharmaceutically relevant ketones (compounds 1, 2, 4, 5 and 7 from Table 4) along with previously catalyzed compounds (compounds 3, 6 and 8 from Table 2) were docked to predict substrate binding and enantiomer production. The binding orientations projected for four out of eight substrate molecules are shown in Fig. 4. Dock poses revealed that the carbonyl functional group connected to the carbon atoms on either side prefers to form hydrogen bond interactions with two (S127 and Y165) out of three catalytic triad residues. The carbonyl oxygen of compound 1 interacts with catalytic residues and the aromatic portion is positioned perpendicular to nicotinamide moiety of NADPH (Fig. 4a). In such an orientation, methylene dioxyphenyl ring forms hydrophobic interactions with substrate binding pocket residues such as Y197, F214 as well as the catalytic residue Y165. In the case of compound 2, the carbonyl group present on pyrolidinone ring interacts with catalytic residues while the phenyl ring of CBZ group forms hydrophobic interactions with surrounding residues (Fig. 4b). The carbonyl group of substituted acetophenone (Fig. 4c, d) establishes hydrophilic interactions similar to compound 1 and 2 whereas the meta-substituted phenyl ring forms hydrophobic interactions. The docking results of the remaining compounds were similar to the above observations.

**Table 4:** Comparison of whole-cell conversion and chemical synthesis of chiral alcohols and their pharmaceutical importance

	Pharmaceutical intermediate structure	Whole-cell catalysis				Chemical synthesis	
		% Product (A)*	% Product (B)**	(*S*) %EE (A)*	(*S*) %EE (B)**	In-house Chemically synthesized compounds	Product and Pharmaceutical use
1	3,4-Methylenedioxyphenyl acetone	95	90	90	82	(*S*)-2-methyloxirane and n-Butyl Lithium [49]	Talampanel Treatment of Epilepsy. Non-competitive antagonist against glutamate receptor [50]
2	1-*N*-carbobenzoxy-3-pyrrolidone	96.8	96	97	50	Sodium borohydride catalyst [51]	Barnidipine Calcium Channel Blocker [52]
3	1-(3,5-Bis-trifluoromethyl-phenyl)-ethanone	23.8	77	100	44	DIP chloride used, difficult chemistry in large scale [53]	Aprepitant Treating chemotherapy induced vomiting. Blocks NK1 receptor [54]
4	1- (2,6-Dichloro-3-fluoro-phenyl)-ethanone	40.2	40	100	92	Ruthenium metal based Naud’s catalyst [55]	Crizotinib Anti-cancer drug, an anaplastic lymphoma kinase inhibitor [56]
5	3-Trifluoromethyl acetophenone	91.1	91	100	91	Asymmetric hydrogenation of keto substrate using Noyori’s ruthenium (II) catalyst [57]	MA-20565 Agricultural Broad spectrum fungicide [58]
6	2-Phenyl-1-thiazol-2-yl-ethanone	100	100	96	89.3	Asymmetric hydrogenation of keto substrate using ligated copper hydride in presence of polymethyl hydrosiloxane [59]	Dolastatin Anti-tumor agent [60]
7	1- (3-methoxyphenyl) ethanone	97.5	97	98	98	Asymmetric hydrogenation of keto substrate using ruthenium complexes [61, 62]	Rivastigmine Treatment of moderate dementia related to Parkinson’s and Alzheimer’s disease [63]
8	3-Oxo-4- (2,4,5-trifluoro-phenyl)-butyric acid methyl ester	98.3	98	46	99	Asymmetric hydrogenation is done by using Adam’s catalyst which is very expensive [48]	Sitagliptin Used for treating diabetes mellitus type 2 [64]

Ketoreduction of key pharmaceutical intermediates after in silico predictions using pET28-ZRK *E.* *coli* with reaction time of 16 h and substrate loading of 0.5 g/L. (*S*)  %EE = (*S*)-specific enantiomeric excess

*(A) = biomass from 100 ml culture and **(B) = biomass from 500 ml culture

#### Molecular dynamics simulations confirm the docking evaluation

We performed molecular dynamics simulations and binding pose of compound 1 (Fig. 4a) with modeled ZRK. The NADPH molecule coordinates acquired from the template structure by superposition method are included in ZRK-compound 1 complex simulation. The MD simulation analyses such as RMSD, interaction energies with cofactor and substrate as well as hydrogen bonding analyses (Additional file 1: Fig. S1) revealed that docking predicted binding mode is reasonable. The interaction pattern of substrates with catalytic residues S127 and Y165 established in the current study was consistent with earlier report [47] which is a prerequisite for further steps of the catalytic mechanism. Time-dependent backbone RMSD showed that the simulations were stable with no observable artifacts. The average RMSD value for ZRK+ NADPH+ compound 1 complex oscillated around 0.2 nm (Additional file 1: Fig. S1). Further, inter-molecular hydrogen bond interaction analyses between ZRK and NADPH revealed 10–12 hydrogen bond interactions were possible and consistent throughout 10 ns simulation. The two hydrogen bonds predicted by molecular docking between ZRK and substrate molecule (compound 1) were also preserved during the time course of simulation (Additional file 1: Fig. S1b). The short range (SR) columbic and Vander Waals interaction energies are calculated (Additional file 1: Fig. S1c, d) for substrate and NADPH with ZRK. The analyses also support the stability of the simulations and reveal that NADPH interacts firmly with ZRK than the substrate. This is anticipated as NADPH makes large surface area contact with ZRK comprising of charged (predominantly) and hydrophobic portions.

Molecular editor tools incorporated in Maestro suite were utilized to edit the carbonyl of compound 1 initially to methylene (CH2) as shown in Additional file 1: Fig. S2b. Hydroxyl groups were substituted at methylene hydrogen atoms to generate models of enantiomeric alcohols (see Additional file 1: Fig. S2c, d). The NADPH is replaced with NADP^+^ for better understanding and visual inspections. The hydroxyl that faces NADP^+^ has S configuration [S–OH] and hydroxyl away from cofactor, facing A129 residue has R-configuration. The hydroxyl group in R-configuration does not have enough room and is sterically crowded; it may lead to a steric clash with A129, Y165 and hydroxyl group of S127. In the case of alternative (*S*)–stereomer, hydroxyl group has enough room to adjust as the location is open as shown in Additional file 1: Fig. S2c. This could be the reason for the substrates (compound 1–7) evaluated in the current study to yield higher amounts of (*S*)-configuration products. Binding orientations of the substrate revealed that the re-face of the substrate toward NADPH results in the formation of the (*S*)-enantiomer of the alcohols for all of the studied compounds except compound 8. Docking studies of compound 1–7 clearly demonstrated that the substrate binding orientations are pro-(S)/Si and consistent with other ketoreductases that yield (*S*)-enantiomers. It is well-known that the stereo-specificity of the enzyme-catalyzed keto reductions can be predicted with Prelog’s rules, which depends on the steric situation of substrates [48]. Noticeably, the steric terms of the substrate with catalytic site residues and NADPH dictate the orientation and further stereo-selectivity of the product. Observations of our current results revealed that NADPH cofactor is bound in the syn conformation, which exposes the Si hydrogen atom of reduced nicotinamide toward the enzyme active site as a reactant for ketone reduction [3]. This understanding helped in generating ~ 90% (*S*)-enantiomeric excess of other important pro-pharmaceutical ketones. It is interesting to note that Sitagliptin ketone intermediate (compound 8) did not concur with the predictions. All the compounds except the compound 8 [3-Oxo-4-(2,4,5-trifluoro-phenyl)-butyric acid methyl ester] yielded (*S*)-enantiomers. Examination of the binding orientation of compound 8 revealed the possibility of variant binding modes (see Additional file 1: Fig. S3a & S3c). The compound 8 binding orientation is like compound 1 with the hydroxyl group towards NADPH but is in R-configuration which is contrary to the orientation of the other compounds. This difference might be arising due to differences in chemical structure, for instance, flexible linkers connected to the carbonyl group (reaction center). In the case of compound 3, the product of commercial interest is the (*R*)-enantiomer but the enzyme converts it to an S-alcohol as predicted by the modelling study.

### Evaluation of the in silico results by using ZRK expressing cells

**Fig. 5:**
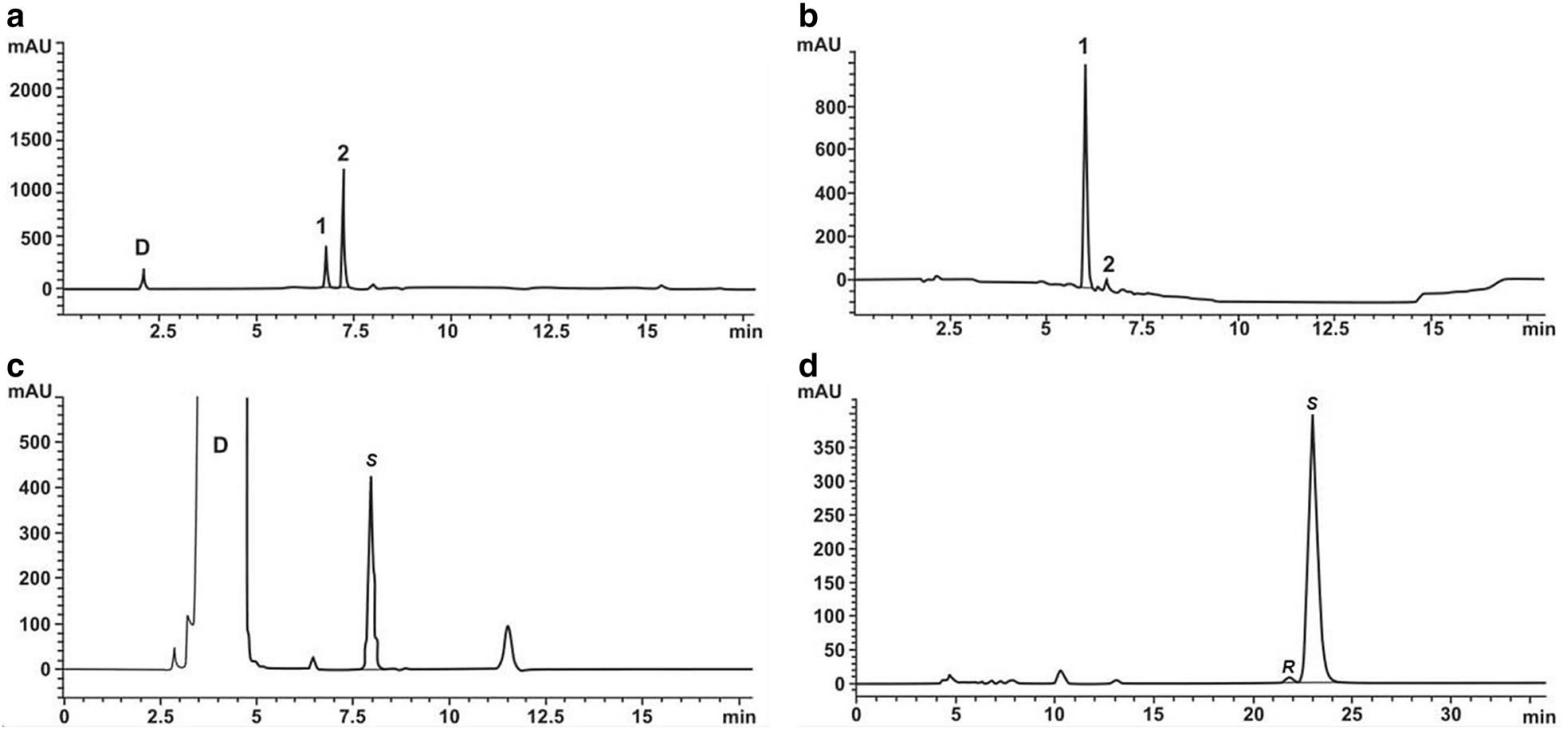
Chromatograms depicting bioconversion of pro chiral ketones. HPLC analysis of whole-cell transformed Crizotinib ketone intermediate (**a**) and the chiral separation of its alcohol (**b**). Similarly, whole-cell transformed dolastatin ketone intermediate (**c**) and the chiral separation of its alcohol (**d**). 1: alcohol product, 2: substrate, D: DMSO, S: (*S*)–isomer and R: (*R*)–isomer. In both the cases, the (*S*)–isomer production is > 90%

A 500 ml scale ketoreduction reaction as outlined in the experimental section was set up for 8 substrates (compounds 1–8 from Table 4) [48–64] that are key intermediates in the syntheses of different therapeutic molecules. These trials were used to isolate the alcohol product in sufficient amounts and carry out a preparative chiral HPLC and determine the nature of the isomer produced. We observe that the (*S*)-enantiomer is preferentially produced during ketoreduction by ZRK and the yields are listed again in Table 4. Chromatograms for Crizotinib and Dolastatin intermediates featuring (*S*)-enantiomeric yield are shown in Fig. 5. Chromatograms for the rest of the substrates are provided in Additional file 1: Fig. S5–S28. All the compounds except the Sitagliptin ketone intermediate (compound 8) yielded (*S*)-enantiomers.

## Conclusions

Amongst the six evaluated ketoreductases, ZRK converts a diverse range of pharmaceutically important prochiral ketones. The mechanism contributing to enantioselectivity towards the production of (*S*)-alcohols has been elucidated by docking and MD simulations with a focus on active site architecture. The computational protocol optimized here can be useful to predict novel substrates that can bind to ZRK and undergo ketone to alcohol conversion. Regeneration of cofactors for biotransformation is usually enabled by either substrate coupled or enzyme coupled reactions [65]. To achieve similar results we used nutrient modulation, cofactor enhancement was attained by switching the carbon source from glucose to sorbitol. It eliminates the need for co-expressing another enzyme that impacts the energy resources of the cell, the same would now be available for chiral conversion. This understanding of the catalytic mechanism and cofactor augmentation can be extended to other ketoreductases and substrates to reduce the enzyme screening space and enable manufacture of high value chiral alcohols.

## Methods

### Reagents and chemicals

*Escherichia coli* strains DH5α (Novagen, U SA) and BL21(DE3) (Novagen, USA) are used as hosts for sub-cloning and over-expression respectively. Restriction enzymes NdeI and XhoI are purchased from NEB (New England Biolabs). A set of high value prochiral ketone intermediates namely, 3,4-methylenedioxyphenyl acetone, 1-N-carbobenzoxy-3-pyrrolidione or 3-hydroxy-pyrrolidone-1-carboxylicacid benzyl ester, 1-(3,5-bis-trifluoromethyl-phenyl)-ethanone, 1- (2,6-dichloro-3-fluoro-phenyl)-ethanone, 3-trifluoromethyl acetophenone, 2-phenyl-1-thiazol-2-yl-ethanone, 1- (3-methoxyphenyl) ethanone, 3-oxo-4- (2,4,5-trifluoro-phenyl)-butyric acid methyl ester as important intermediates in various pharmaceutical syntheses are synthesized as outlined in Additional file 1. Corresponding racemic alcohols are synthesized from respective ketones using the sodium borohydride method reported elsewhere [49].

### Construction of the different ketoreductase expression vectors and transformation into BL21(DE3)

Genes encoding the six enzymes from diverse taxa i.e., *Sulfolobus* *sulfotaricus* alcohol dehydrogenase (ADH) from archaea (Ssadh), fungal SDRs *Zygosaccharomyces rouxii* SDR (ZRK) and Hansenula polymorpha DL-1 peroxisomal 2,4-dienoyl-CoA reductase (Hketo) and lastly, *Corynebacterium* strain ST-10 phenylacetaldehyde reductase (PAR), *Synechococcus *sp. PCC 7942 3-ketoacyl-[acyl-carrier-protein] reductase (FabG) and *Bacillus *sp. ECU0013 ADH (ByueD) from bacteria are synthesized using optimal codon usage for *E. coli* (Geneart, http://www.geneart.com). The gene sequences are deposited in DDBJ with the following accession numbers (LC325171 for SsADH, LC325172 for ZRK, LC325173 for PAR, LC325174 for ByeuD, LC325175 for Hketo, LC325176 for FabG). These are then sub-cloned into pET28a vector (Novagen) using the restriction sites NdeI and XhoI with an N-terminal His-tag in frame. Soluble protein expression experiments are performed as in the protocol followed for whole-cell transformation. The cells are harvested 18 h post induction and the soluble His-tagged ketoreductases are purified by Ni–NTA affinity chromatography.

### Whole-cell biotransformation using *E. coli* expressing various ketoreductases

Over-expression of the enzymes using the different pET28a-ketoreductase plasmids is done in *E. coli* BL21(DE3) strain using standard procedures [66]. In brief, the pET28a-ketoreductase plasmid transformed BL21 (DE3) cells are grown in 100 ml LB-broth with 50 μg/ml Kanamycin at 37 °C to an OD of 0.6, followed by induction with 200 μM IPTG and continued incubation at 18 °C for 16 h. The cells are harvested and resuspended in 10 ml phosphate buffered saline (PBS) containing 10% carbon source (w/v), either phosphate buffered saline (PBS), PBS + glucose or PBS + sorbitol. The cells are acclimatized for 2 h to use the provided carbon source before adding the prochiral ketone substrates to the suspension at 0.5 g/L concentration. This culture and ketone suspension is incubated at 30 °C for 16 h to allow the cells to perform the biotransformation. To determine the chiral alcohol yield and EE %, the cell suspension is extracted with an equal volume of Ethyl acetate and the organic layer is subjected to HPLC analysis. *E. coli* BL21(DE3) cells over-expressing the different ketoreductases are grown overnight at 37 °C in 500 ml LB followed by induction with IPTG and incubation as above. The cells are harvested and resuspended in 500 ml of PBS with 10% sorbitol (w/v) before continuing the above procedure. This is done to compare the consistency in production of chiral synthons by ZRK at a higher volume.

### Analytical methods

The conversion and enantiomeric excess of the whole-cell biotransformation is monitored using HPLC. A Shimadzu LC-2010CHT HPLC equipped with Photodiode Array detector set at 205 nm is utilized. Either acetonitrile and 0.1% trifluoroacetic acid or acetonitrile and 0.05% perchloric acid in water is delivered to a 150 mm X 4.6 mm, 5 μm, Zorbax SB-CN, column at a rate of 1 mL/min. Normal phase HPLC is carried out to determine the enantiomeric excess of the isomers produced. The mobile phase, 2% n-hexane and 98% *n*-hexane: ethanol (50:50) is delivered to a Chiralcel OZ-H 4.6 X 250 mm, 5 µm, at a rate of 0.7 mL/min. The racemic mixture generated by sodium borohydride method (from the prochiral ketones) is separated into (*R*) or (*S*) isomers by using preparative chiral HPLC techniques and their retention time (RT) value is fixed as a standard for determining the enantiomeric excess of the isomers produced following the ketoreduction.

### Homology model generation for ZRK

*Zygosaccharomyces rouxii* SDR (ZRK: Swiss-Prot code Q9UV57 homologs are identified using Protein BLAST and all non-redundant proteins as search set on the NCBI database. The best hit is used as a template for homology modelling using SWISS-MODEL webserver [67]. The template structure selected by blastp search is specified explicitly to generate homology models which are validated by PROCHECK software [68, 69].

### Binding mode determination of substrate molecules by molecular docking

Eight pro-chiral pharmaceutically important substrates (Nos. 1–8 from Table 4) are considered in catalytic site docking to determine binding orientations. The ligands are converted to 3D using LigPrep of Schrodinger suite which performs tasks such as protonation at specified pH, generation of tautomers, ionization states, and probable stereomers [70]. The output is considered for multi-conformation generation by Macromodel [71] module which uses Monte Carlo method of exploring torsional space. OPLS_2005 force field is used and energy minimization is done in 500 steps using TNCG method (Energy window is 21 kJ/mol which is default value). The redundancy threshold is 0.5 Å RMSD, and redundant conformers are removed. The maximum number of conformers to be generated is set to 25. Default parameters are used for the remaining options. Glide v5.5 docking module (in Schrodinger suite) is employed to predict the binding orientations for the eight substrates molecules [72]. Glide-SP algorithm uses pre-computed grids generated using receptor sites defined by the centroids of the bound ligands/selected atoms. The lowest energy poses are then subjected to a Monte Carlo procedure that sample nearby torsional minima. The compounds are then ranked using GlideScore that includes terms for steric clashes and buried polar groups. Default van der Waal’s scaling is used (1.0 for the receptor and 0.8 for the ligand). Advanced settings are edited to increase the pose sampling. Total 10,000 poses (default 5000) per ligand are set for the initial phase of docking and poses per ligand per energy minimization raised to 1000 from 400. Total ten poses per ligand are saved as output for post docking analyses.

### Molecular dynamics simulations

MD simulations are performed using GROMACS program (version 4.5.4) with the Amber99SB force field [73]. AM1-BCC charges are computed for substrates with the Antechamber software [74]. For MD simulations, each system is inserted in a water box (pH 7.0) of TIP3P water, which extended at least 12 Å away from any given protein atom. All systems are neutralized by adding counter ions and replacing the overlapping solvent molecules. Steepest descent algorithm is used for energy minimization. Position restrained MD run performed for 200 ps during heating step (300 K) so that solute molecules (protein and ligand) molecules are restrained whereas water molecules with counter ions are allowed to move and equilibrate. The final simulation is performed for 10 ns with a 2-fs time step. Temperature and pressure are maintained at 300 K and 1 atm respectively using the v-rescale temperature and Parrinello–Rahman pressure coupling method.

## Additional file

**Additional file 1.** Supplementary Information.

## Abbreviations

*E. coli*: *Escherichia coli*
HPLC: high-performance liquid chromatography
OD_600_: optical density at the wavelength of 600 nm
%EE: enantiomeric excess percentage
RMSD: Root Mean Square Deviation
PBS: phosphate buffered saline
NAD: Nicotinamide Adenine Dinucleotide
NADP: Nicotinamide Adenine Dinucleotide Phosphate
MD: molecular dynamics
