# Supplementary material for: A strategy to identify a ketoreductase that preferentially synthesizes pharmaceutically relevant (S)-alcohols using whole-cell biotransformation
Source: Microb Cell Fact. 2018 Dec 3;17:192. doi: 10.1186/s12934-018-1036-2 (PMC6276252; doi:10.1186/s12934-018-1036-2)
Supplement: Supplementary file 1 — Additional file 1. Supplementary Information. [file 12934_2018_1036_MOESM1_ESM.doc]

Supplementary Information for

**A strategy to identify a ketoreductase that preferentially synthesizes pharmaceutically relevant (*S*)-alcohols using whole-cell biotransformation**

Saiful F Haq†^1^, Anirudh P Shanbhag†^2, 7^, Subbulakshmi Karthikeyan^1,3^, Imran Hassan^1,8^, Kannan Thanukrishnan^1,4^, Abhishek Ashok^1^, Sunilkumar Sukumaran^1^, Ramaswamy S^5^, Nagakumar Bharatham^6^, Santanu Datta^2^, Shalaka Samant^1^, Nainesh Katagihallimath^2*^

^1^Anthem Biosciences Pvt. Ltd., Bengaluru, India.

^2^Bugworks Research Pvt. Ltd., Bengaluru, India.

^3^Centre for Pharmaceutical Biotechnology, University of Illinois Chicago.

^4^Shasun Research Center, Chennai, India.

^5^Institute for Stem Cell Biology and Regenerative Medicine, NCBS, Bengaluru, India.

^6^Centre for Cellular and Molecular Platforms, NCBS, Bengaluru, India.

^7^Department of Biophysics, Molecular Biology and Bioinformatics, University of Calcutta, Kolkata, India.

^8^PerkinElmer, Bengaluru, India

* Email: [nainesh@bugworksresearch.com,](mailto:nainesh@bugworksresearch.com,) Tel: +91 80 67185247

^†^ S.F.H and A.P.S contributed equally to this work.

**Supplementary methods:**

**Chemical synthesis of prochiral ketones and their corresponding racemic alcohols:**

1. **Synthesis of 3,4-methylenedioxyphenyl acetone (compound 1):**

**
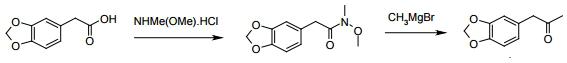
**

**Step-1: Preparation of 2-Benzo[1,3]dioxol-5-yl-N-methoxy-N-methyl-acetamide**

To a solution of 3,4- (Methylenedioxy)phenylacetic acid (2 g, 0.011 mol) in dichloromethane (30 mL) was added EDCI.HCl (4.24 g, 0.022 mol), HOBt (0.75 g, 0.006 mol), DIPEA (3.9 mL, 0.022 mol) and N,O-dimethylhydroxylamine hydrochloride (1.4 g, 0.014 mol) at 0 °C and stirred at room temperature for 18 h. It was diluted with water (50 mL) and concentrated to remove DIPEA and dichloromethane. The resultant aqueous layer was extracted with ethyl acetate (2 x 50 mL). The combined organic layer was washed with water and brine solution, dried over anhydrous Na2SO4 and concentrated under reduced pressure. The crude material was purified by column chromatography to get 2-benzo[1,3]dioxol-5-yl-N-methoxy-N-methyl-acetamide as a brown solid. Yield 1.8 g (73 %)

**Step-2: Preparation of 1-Benzo[1,3]dioxol-5-yl-propan-2-one**

To a solution of 2-benzo[1,3]dioxol-5-yl-N-methoxy-N-methyl-acetamide (1.6 g, 1 mol) in THF (15 mL) was added methylmagnesium bromide dropwise at 0 °C . The reaction mixture was brought to room temperature and stirred at room temperature for 2 h. The reaction mixture was quenched with saturated ammonium chloride solution and extracted with ethyl acetate (2 x 50 mL). The combined organic layer was washed with water and brine solution, dried over anhydrous Na2SO4 and concentrated under reduced pressure. The crude material was purified by column chromatography to afford 1-benzo[1,3]dioxol-5-yl-propan-2-one as a pale yellow liquid. Yield 0.7 g (55 %)

1. **Synthesis of 2-Phenyl-1-thiazol-2-yl-ethanone (compound 6)**

**
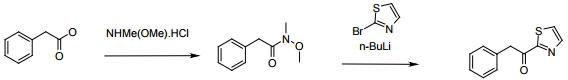
**

**Step-1: Preparation of N-Methoxy-N-methyl-2-phenyl-acetamide**

To a solution of phenylacetic acid (5 g, 0.037 mol) in dichloromethane (60 mL) was added EDCI.HCl (13.9 g, 0.073 mol), HOBt (4.64 g, 0.047 mol), DIPEA (12.7 mL, 0.018 mol) and N,O-dimethylhydroxylamine hydrochloride (4.64 g, 0.048 mol) at 0 °C and stirred at room temperature for 18 h. It was diluted with water (150 mL) and concentrated to remove DIPEA and dichloromethane. The resultant aqueous layer was extracted with ethyl acetate (2 x 100 mL). The combined organic layer was washed with water and brine solution, dried over anhydrous Na2SO4 and concentrated under reduced pressure. The crude material was purified by column chromatography to give rise to N-methoxy-N-methyl-2-phenyl-acetamide as brown solid. Yield 3.7 g (56 %)

**Step-2: Preparation of 2-Phenyl-1-thiazol-2-yl-ethanone**

A solution of n-butyllithium (1.6 M in hexane, 5.82 mL, 1.2eq) and TMEDA (2 mL, 0.013 mol) was taken in THF (20 mL) and cooled at -78 °C. A solution of 2-bromothiazole (1.2 mL, 0.013 mol) was added dropwise and stirred at -78 °C for 2 h. To this mixture was added a solution N-methoxy-N-methyl-2-phenyl-acetamide (2 g, 0.011 mol) in THF and stirred at -78 °C for 1 h. The reaction mixture was brought to -10 °C and stirred for 2 h. It was quenched with saturated potassium hydrogensulfate and extracted with dichloromethane (2 x 50 mL). The combined organic layer was washed with water and brine solution, dried over anhydrous Na2SO4 and concentrated under reduced pressure. The crude material was purified by column chromatography to get 2-phenyl-1-thiazol-2-yl-ethanone as yellow solid. Yield 1.2 g (53 %)

1. **Synthesis of Sitagliptin-Ketone intermediate9 and the corresponding alcohol (compound 8)**

**
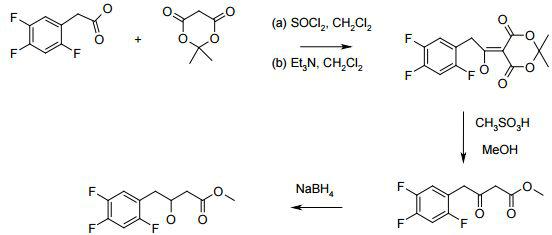
**

Step-1: Synthesis of 5-[1-Hydroxy-2- (2,4,5-trifluoro-phenyl) -ethylidene] -2,2-dimethyl- [1,3] dioxane -4,6-dione:

To a solution of 2, 4, 5-trifluorophenylacetic acid (4 g, 0.021 mol) in dichloromethane (40 mL) was added dimethyl formamide (0.3 mL) and stirred for 15 min. Thionyl chloride (1.84 mL, 0.025 mol) was added to the reaction mixture at 0 °C and stirred for 3 h at 45 °C. The solvent was removed under nitrogen atmosphere to afford acid chloride. It was used as such without further purification. To the acid chloride in dichloromethane (20 mL) was added Meldrum's acid (3.63 g, 0.025 mol) in dichloromethane (20 mL) followed by triethylamine (5.9 mL, 0.042 mol) dropwise at -5 °C and stirred for 3 h at the same temperature. The reaction mixture was quenched with water (50 mL) and extracted with dichloromethane (2 x 100 mL). The combined organic layers were washed with saturated sodium bicarbonate solution (25 mL), water (2 x 10 mL) and saturated brine solution (50 mL), dried over anhydrous Na_2_SO4, filtered and concentrated under reduced pressure. To the crude compound at -5 °C, aqueous sodium hydroxide (3 g, in 25 mL water) was added and stirred for 2 h at room temperature. The solid precipitated solid was filtered and dried under reduced pressure. It was triturated with ethylacetate/hexane solution (1:1) to afford 5-[1-Hydroxy-2-(2,4,5-trifluoro-phenyl)-ethylidene]-2,2-dimethyl-[1,3]dioxane-4,6-dione as a pale yellow solid. Yield 3.8 g (57 %)

Step-2: Synthesis of 3-Oxo-4- (2,4,5-trifluoro-phenyl)-butyric acid methyl ester

To a suspension of 5-[1-hydroxy-2- (2,4,5-trifluorophenyl)ethylidene]-2,2-dimethyl-1,3-dioxane-4,6-dione (1.5 g, 4.7 mmol) in methanol (15 mL) was added methane sulfonic acid (0.6 mL, 9.5 mmol) at 0 °C. The reaction mixture was stirred at 65 °C for 2 h. The reaction mixture was diluted with water (5mL) and the methanol was removed under reduced pressure. The resultant solution was again diluted with water (30 mL) and extracted with dichloromethane (2 x 100 mL). The combined organic layer

was washed with saturated brine solution (30 mL), dried over anhydrous Na2SO4, filtered and concentrated. The crude material was purified by column chromatography to result in 3-Oxo-4-(2,4,5-trifluoro-phenyl)-butyric acid methyl ester as colorless gummy material. Yield:0.8 g (68 %)

Compound 7 [1]

Compound 5 [2]

Compounds 2, 3 and 4 and the remaining compounds listed in Table 4 were procured from Sigma-Aldrich.

Supplementary results

*In silico* predictions and molecular dynamic simulations


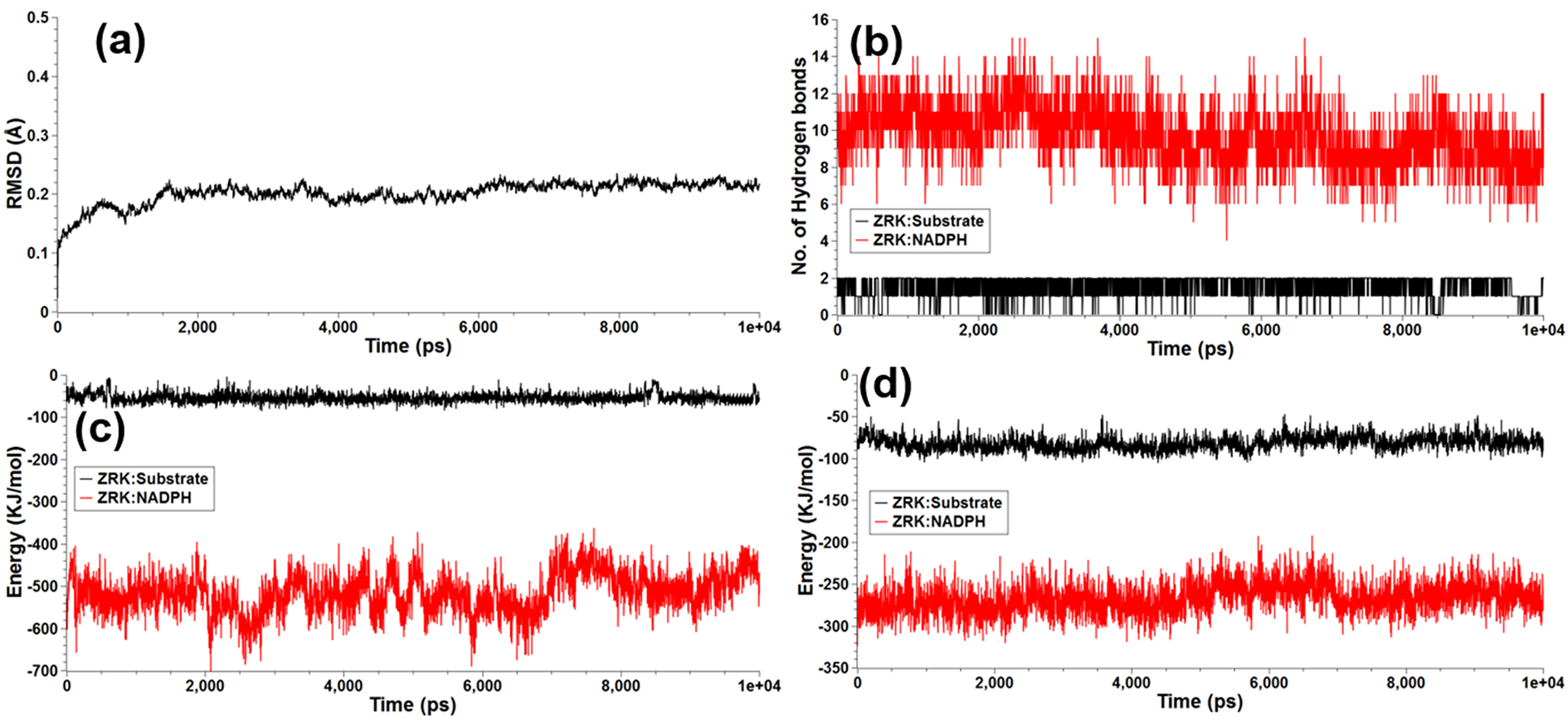


**Figure S1.** MD simulation analyses of compound 1 with ZRK. The root mean square deviation for total 10ns simulation (A), No. of hydrogen bonds (B), columbic interaction energy (C), and vdW interaction energy (D) between ligands and protein were represented. Black lines in B, C, D represent calculations between compound 1 and protein and red lines represent between NADPH and protein.

The MD simulations were judged to be stable as evidenced by the time dependent evaluation of backbone RMSD. Analysis of MD simulation trajectory suggests that the interaction pattern predicted by molecular docking approach is stable and the ZRK structure developed by homology model method is reasonable for further usage. The average RMSD value for ZRK + NADPH + compound 1 complex simulation oscillated around 0.2 nm (Figure S1A) revealed that the system was stabilized thus confirming that the simulation is reliable with no observable discrepancies. Further, inter-molecular hydrogen bond interaction analyses between ZRK and NADPH revealed 10 to 12 hydrogen bond interactions are possible and consistent throughout 10 ns simulation. The two hydrogen bonds predicted by molecular docking between ZRK and substrate molecule (compound 1) were also preserved during the time course of simulation (Figure S1B). The short range (SR) columbic and Vander Waals (VDW) interaction energies were calculated (Figure S1C & S1D) for substrate and NADPH with ZRK. These analyses also support the stability of the simulations and reveal that NADPH interacts firmly with ZRK than substrate. This is anticipated as NADPH makes large surface area contact with ZRK comprising of charged (predominantly) and hydrophobic portions.


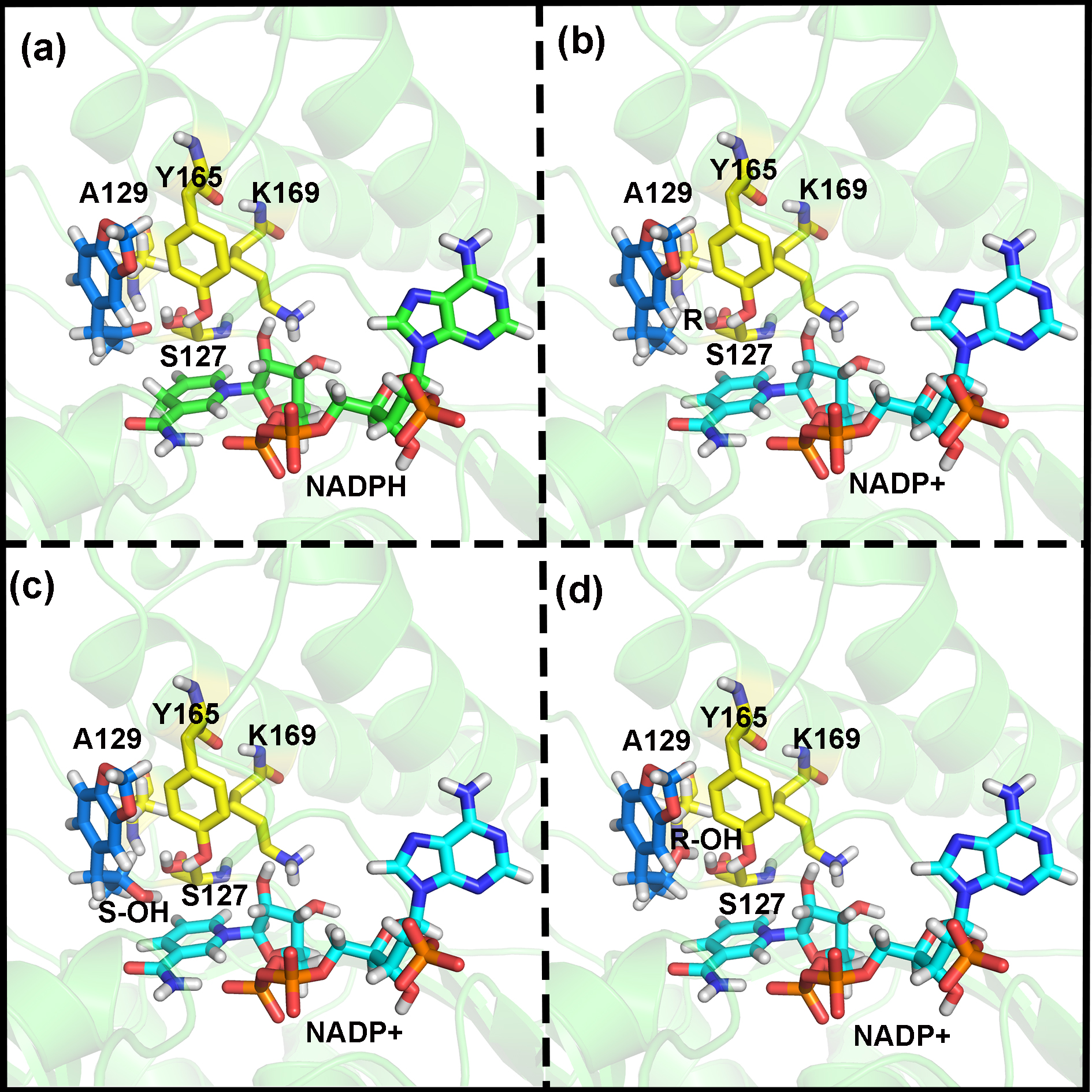


**Figure S2.** Generation of enantiomeric alcohols by molecular editing approach. Dock pose of compound 1 with ZRK (a), compound 1 derivative with methylene in place of carbonyl (b), addition of S- (c) and R- (d) alcohol groups to represent their respective positions. NADPH and NADP+ depicted in green and cyan sticks, respectively. Catalytic triad residues highlighted with yellow sticks whereas compound 1 shown as blue sticks.


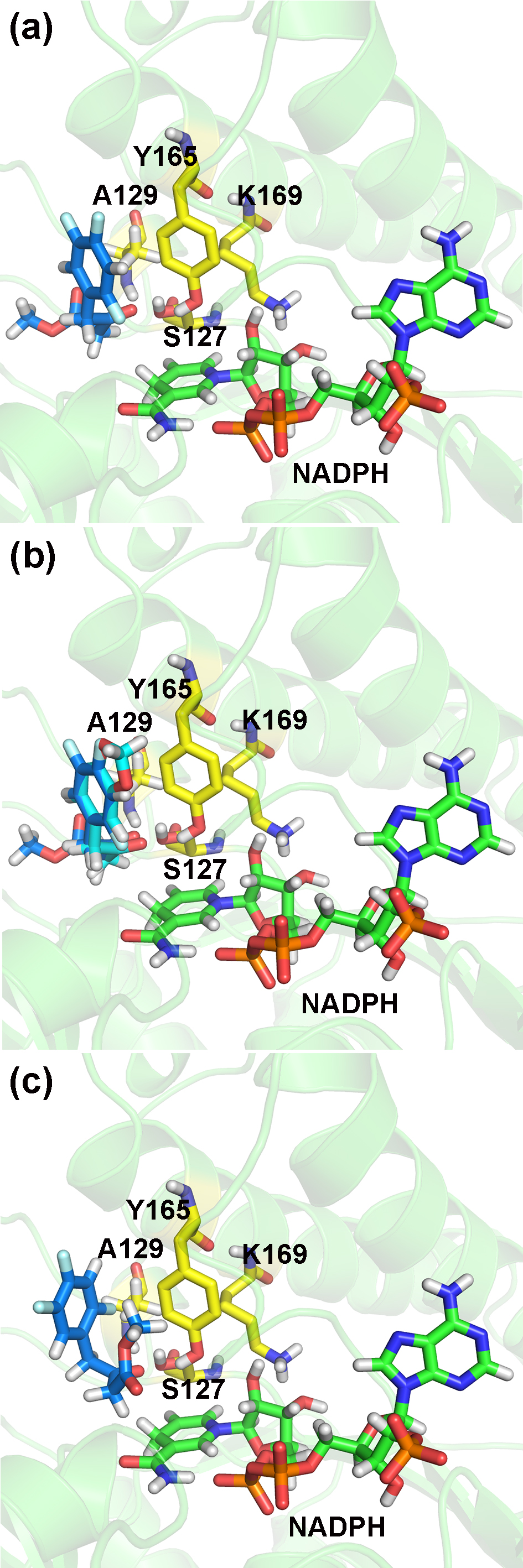


**Figure: S3.** Variant binding modes of compound 8 with ZRK catalytic site. Binding mode 1 with ZRK (a), superposition of compound 8 on compound 1 docking orientation (b) and second binding orientation of compound 8 (c). NADPH depicted in green and catalytic triad residues highlighted with yellow sticks. The compounds 8 and 1 are shown as blue and cyan sticks, respectively.

**Complete Gel Picture**

**
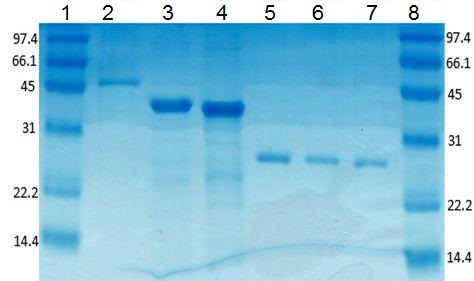
**

**Figure S4.** SDS PAGE of all the isolated ketoreductases. Lanes 1: Phenyl acetaldeyhyde reductase (PAR), 2. *Sulfolobus sulfotaricus* alcohol dehydrogenase (Ssadh), 3. *Zygosaccharomyces rouxii* SDR (ZRK), 4. *Hansenula polymorpha* ketoreductase (Hketo), 5. *Bacillus subtilis* yueD (ByueD), 6. 3-oxoacyl-[acyl-carrier-protein] reductase (FabG) & 7: Low range molecular weight marker

**Chromatograms depicting the chemically synthesized enantiomers**


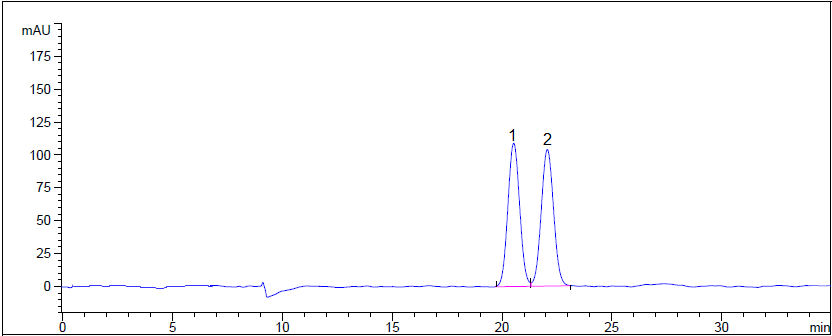


**Figure S5.** Chromatogram depicting the seperation of the chemically synthesized 1: (R)-isomer (RT=20.533) 2: *(S)-isomer* (RT=22.064) of Talampanel intermediate alcohol (2,3-Methylenedioxyphenol) solubilized in DMSO (D)


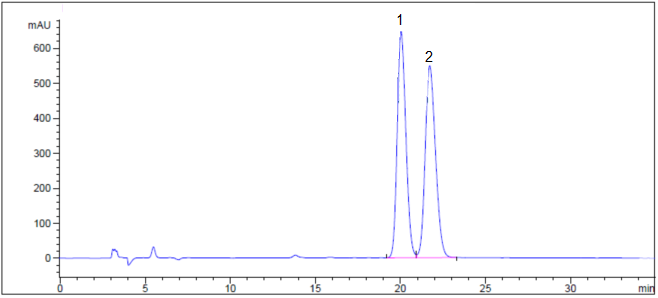


**Figure S6.** Chromatogram depicting the seperation of the chemically synthesized 1: (R)-isomer (RT=20.032) 2: *(S)-isomer* (RT=21.121) of Barnidipine ketone intermediate ( N-CBZ-pyrrolidinone) solubilized in DMSO (D)


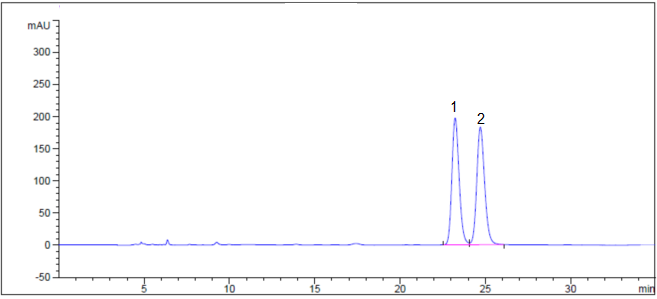


**Figure S7.** Chromatogram depicting the seperation of the chemically synthesized 1: (R)-isomer (RT=23.230) 2: *(S)-isomer* (RT=24.705) of Dolastatin ketone intermediate (2-Phenyl-1-thiazol-2-yl-ethanone)


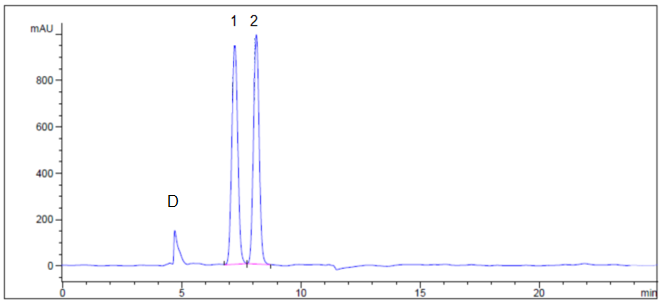


**Figure S8.** Chromatogram depicting the seperation of the chemically synthesized 1: (R)-isomer (RT=7.221) 2: *(S)-isomer* (RT=8.127) of Aprepitant ketone intermediate (1-(3,5-Bis-trifluoromethyl-phenyl)-ethanone solubilized in DMSO (D)


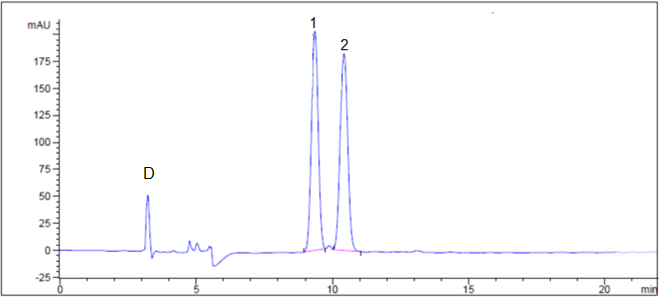


**Figure S9.** Chromatogram depicting the seperation of the chemically synthesized 1: (R)-isomer (RT=9.360) 2: *(S)-isomer* (RT=10.430) of Sitagliptin ketone intermediate (3-Oxo-4- (2,4,5-trifluoro-phenyl)-butyric acid methyl ester) solubilized in DMSO (D)


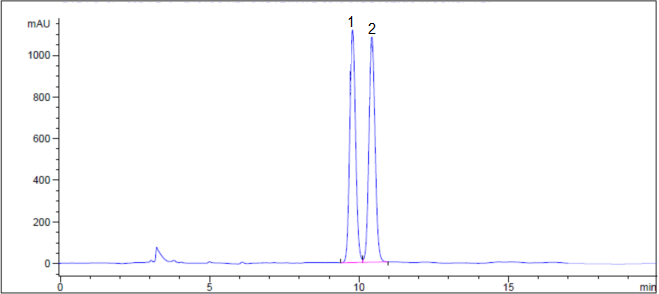


**Figure S10.** Chromatogram depicting the seperation of the chemically synthesized 1: *(S)-isomer* (RT=9.783) 2: (R)-isomer (RT=10.431) of Rivastigmine ketone intermediate (1-(3-methoxyphenyl) ethanone)


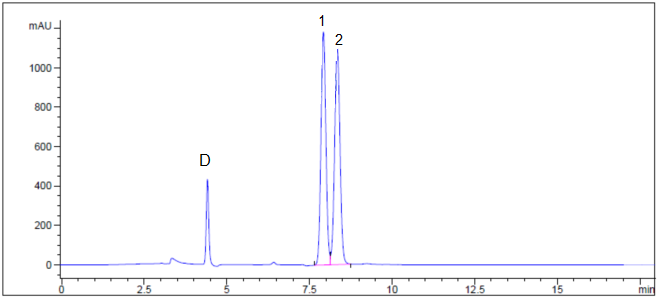


**Figure S11.** Chromatogram depicting the seperation of the chemically synthesized D:DMSO 1: *(S)-isomer* (RT=7.923) 2: (R)-isomer (RT=8.342) of Crizotinib ketone intermediate ( 1-(2,6-Dichloro-3-fluoro-phenyl)-ethanone)


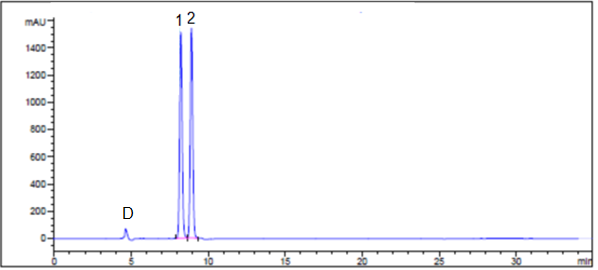


**Figure S12.** Chromatogram depicting the seperation of the chemically synthesized 1: (R)-isomer (RT=7.923) 2: *(S)-isomer* (RT=8.342) of MA-20565 ketone-intermediate (1(3-Trifluoromethyl acetophenone))

**Chromatograms depicting the enantiomeric excess of the alcohols produced through biotransformation**


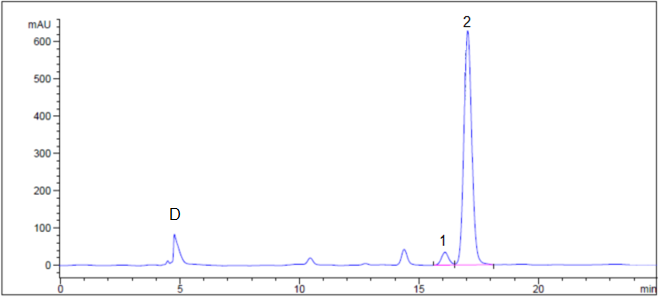


**Figure S13.** Chromatogram depicting the seperation of the peaks 1: (R)-isomer (RT=16.088) 2: *(S)-isomer* (RT=17.040) of Talampanel intermediate alcohol (2,3-Methylenedioxyphenol) after biotransformation solubilized in DMSO (D)


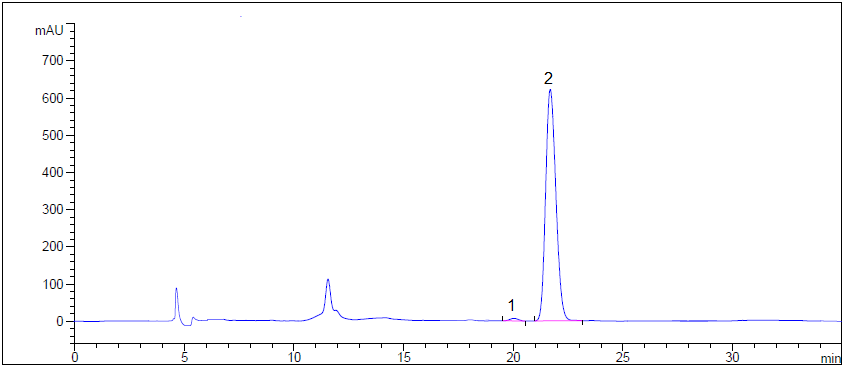


**Figure S14.** Chromatogram depicting the seperation of the peaks 1: (R)-isomer (RT=20.001) 2: *(S)-isomer* (RT=22.101) of Barnidipine ketone intermediate ( N-CBZ-pyrrolidinone) after biotransformation solubilized in DMSO (D)


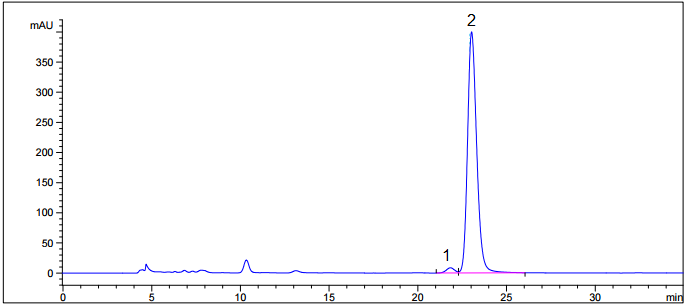


**Figure S15.** Chromatogram depicting the seperation of the peaks 1: (R)-isomer (RT=21.837) 2: *(S)-isomer* (RT=23.607) of Dolastatin ketone intermediate (2-Phenyl-1-thiazol-2-yl-ethanone) after biotransformation solubilized in DMSO (D)


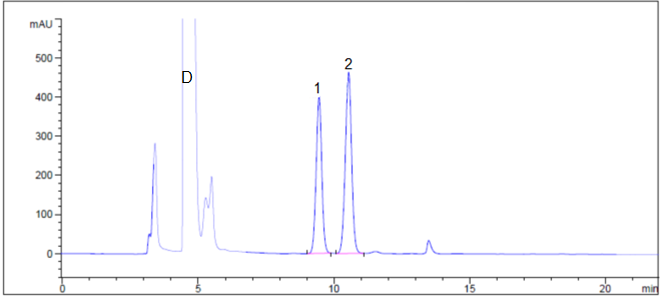


**Figure S16.** Chromatogram depicting the seperation of the peaks 1: (R)-isomer (RT=9.457) and 2: *(S)-isomer* (RT=10.545) of Sitagliptin ketone intermediate (3-Oxo-4- (2,4,5-trifluoro-phenyl)- butyric acid methyl ester) after biotransformation solubilized in DMSO (D)


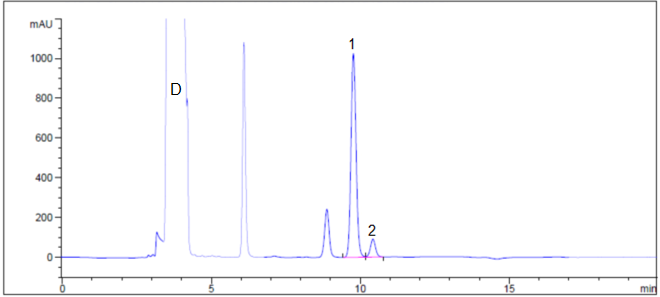


**Figure S17.** Chromatogram depicting the seperation of the peaks 1: *(S)-isomer* (RT=9.770) and 2: (R)-isomer (RT=10.426) of Rivastigmine ketone intermediate (1-(3-methoxyphenyl) ethanone) after biotransformation solubilized in DMSO (D)


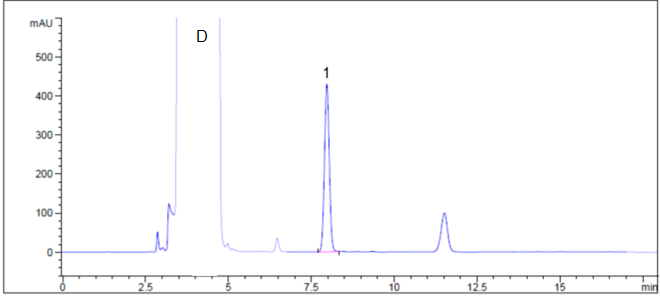


**Figure S18.** Chromatogram depicting the seperation of the peaks 1: *(S)-isomer* (RT=7.971) of Crizotinib ketone intermediate ( 1-(2,6-Dichloro-3-fluoro-phenyl)-ethanone) after biotransformation solubilized in DMSO (D)


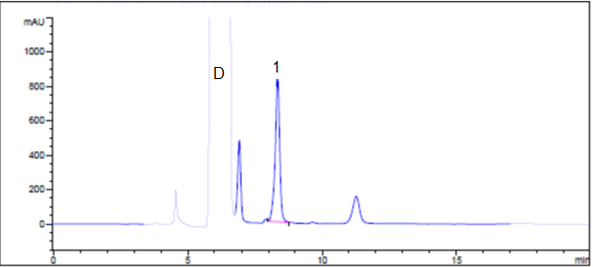


**Figure S19.** Chromatogram depicting the seperation of the peaks 1: *(S)-isomer* (RT=8.947) of MA-20565 ketone-intermediate (1-(3-Trifluoromethyl acetophenone)) after biotransformation solubilized in DMSO (D)

**Chromatograms depicting the biotransformation of ketones**


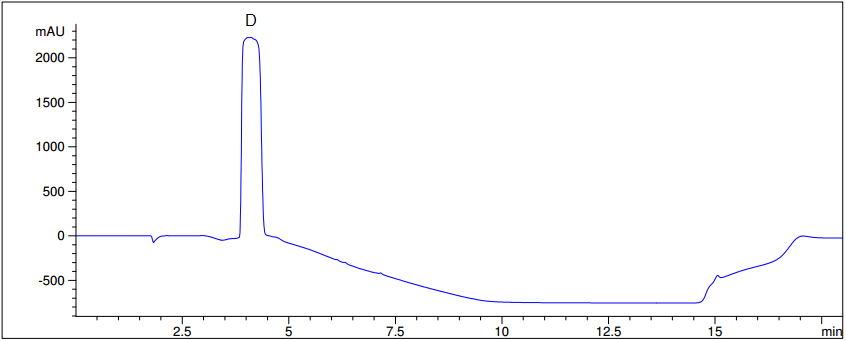


**Figure S20.** Chromatogram depicting a blank run without any substrate/product but with DMSO (D)


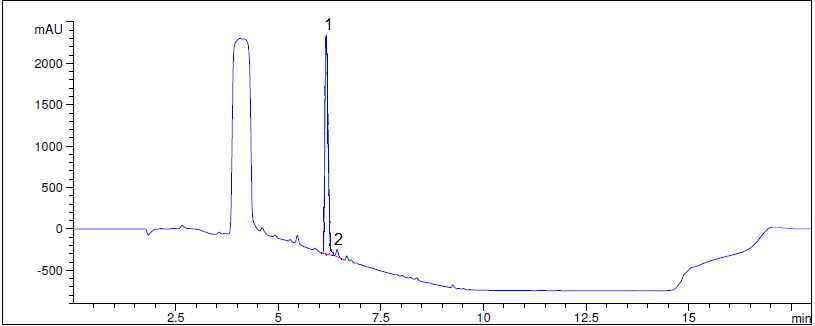


**Figure S21.** Chromatogram depicting the peak D:DMSO 1: Product- Alcohol (RT= 6.129) 2: Substrate- Ketone (RT=6.396) of Talampanel intermediate (2,3-Methylenedioxyphenyl acetone)


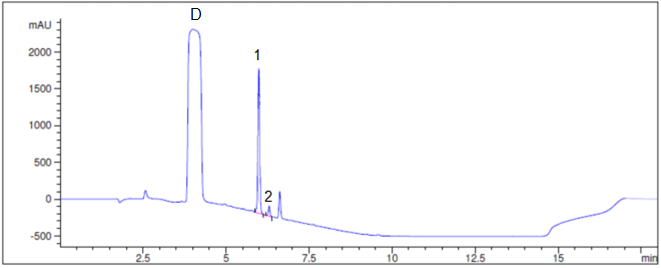


**Figure S22.** Chromatogram depicting the peaks D:DMSO 1: Product- Alcohol (RT= 5.988) 2: Substrate- Ketone (RT=6.301) of Barnidipine ketone intermediate ( N-CBZ-pyrrolidinone)


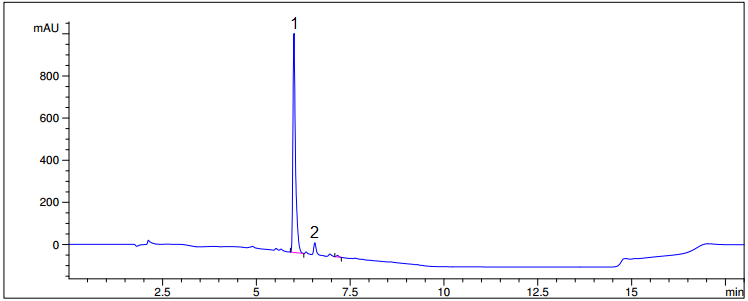


**Figure S23.** Chromatogram depicting the peaks D:DMSO 1: Product- Alcohol (RT= 6.000) 2: Substrate- Ketone (RT=7.162) of Dolastatin ketone intermediate (2-Phenyl-1-thiazol-2-yl-ethanone)


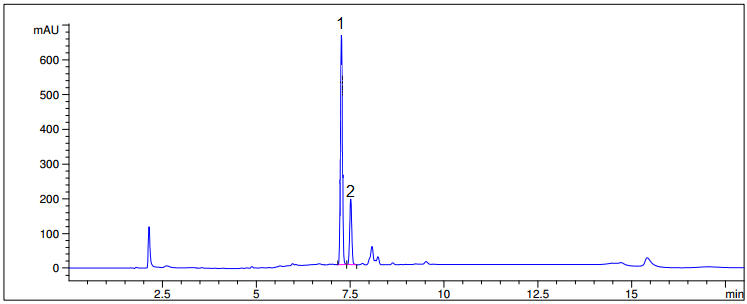


**Figure S24.** Chromatogram depicting the peaks D:DMSO 1: Product- Alcohol (RT= 7.338) 2: Substrate- Ketone (RT=7.586) of Aprepitant ketone intermediate (1-(3,5-Bis-trifluoromethyl-phenyl)-ethanone)


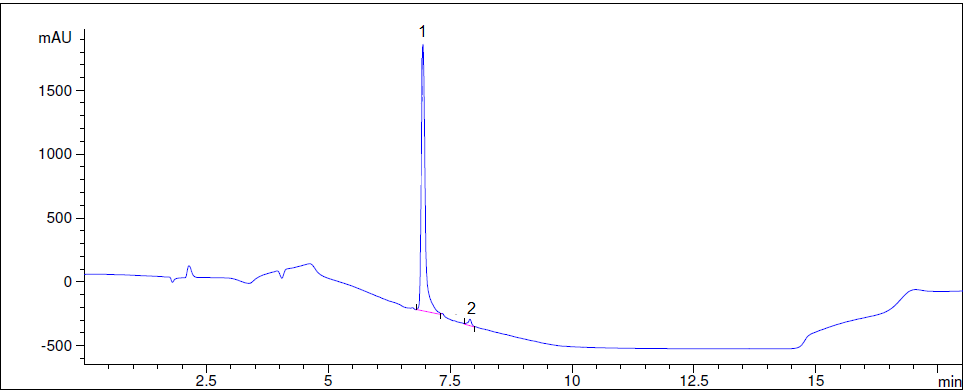


**Figure S25.** Chromatogram depicting the peaks D:DMSO 1: Product- Alcohol (RT= 6.942) 2: Substrate- Ketone (RT=7.910) of Sitagliptin ketone intermediate (3-Oxo-4- (2,4,5-trifluoro-phenyl) -butyric acid methyl-ester)


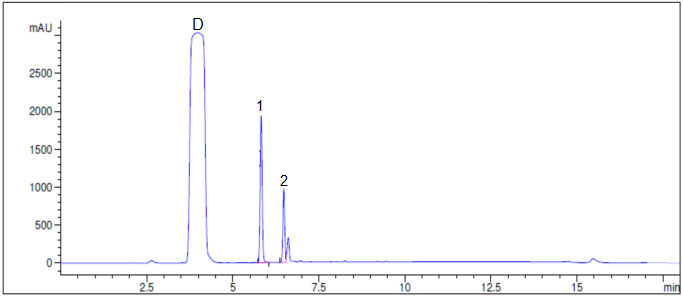


**Figure S26.** Chromatogram depicting the seperation of the peaks D: DMSO 1: Product -alcohol (RT=5.826) and 2: Substrate -ketone (RT=6.483) of Rivastigmine ketone intermediate (1-(3-methoxyphenyl) ethanone)


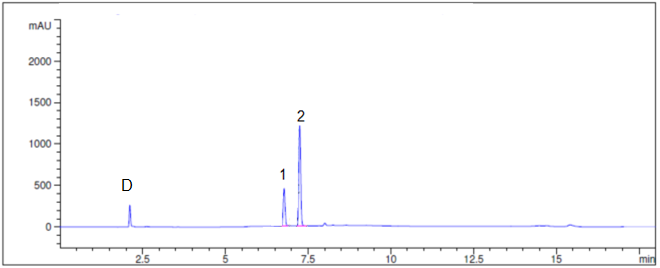


**Figure S27.** Chromatogram depicting the seperation of the D:DMSO 1: Product-alcohol (RT=6.777) and 2: Substrate-ketone (RT=7.249) of Crizotinib ketone intermediate ( 1-(2,6- Dichloro-3-fluoro-phenyl) -ethanone)


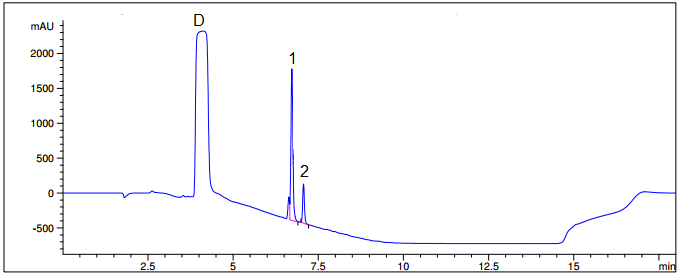


**Figure S28.** Chromatogram depicting the seperation of the D: DMSO 1: Product-alcohol (RT=6.725) and 2: Substrate-ketone (RT=7.067) of MA-20565 ketone-intermediate (1(3-Trifluoromethyl acetophenone))

**References**

1. S. L. Buchwald, B. T. Watson, R. T. Lum and W. A. Nugent, *Journal of the American Chemical* *Society*, 1987, 109, 7137-7141.
2. J. L. Leazer, R. Cvetovich, F.-R. Tsay, U. Dolling, T. Vickery and D. Bachert, *The Journal of* *Organic Chemistry*, 2003, 68, 3695-3698.
